# Novel Short-Course Therapy and Morphism Mapping for Clinical Pulmonary *Mycobacterium kansasii*

**DOI:** 10.1128/AAC.01553-20

**Published:** 2021-04-19

**Authors:** Shashikant Srivastava, Jann-Yuan Wang, Gesham Magombedze, Moti Chapagain, Hung-Ling Huang, Devyani Deshpande, Scott K. Heysell, Jotam G. Pasipanodya, Tawanda Gumbo

**Affiliations:** a Department of Pulmonary Immunology, University of Texas Health Science Center at Tyler, Tyler, Texas, USA; b Center for Infectious Diseases Research and Experimental Therapeutics, Baylor Research Institute, Baylor University Medical Center, Dallas, Texas, USA; c Department of Immunology, UT Southwestern Medical Center, Dallas, Texas, USA; d Department of Internal Medicine, National Taiwan University Hospital, Taipei, Taiwan; e Kaohsiung Municipal Ta-Tung Hospital, Kaohsiung Medical University Hospital, Kaohsiung, Taiwan; f Division of Pulmonary and Critical Care Medicine, Kaohsiung Medical University Hospital, Kaohsiung, Taiwan; g Department of Internal Medicine, Kaohsiung Medical University Hospital, Kaohsiung, Taiwan; h Graduate Institute of Medicine, College of Medicine, Kaohsiung Medical University, Kaohsiung, Taiwan; i Division of Infectious Diseases and International Health, University of Virginia, Charlottesville, Virginia, USA; j Quantitative Preclinical and Clinical Sciences Department, Praedicare, Dallas, Texas, USA; k Praedicare Laboratories, Praedicare, Dallas, Texas, USA

**Keywords:** time to extinction, moxifloxacin, treatment duration, hollow-fiber system, tedizolid

## Abstract

Standard therapy (isoniazid, rifampin, and ethambutol), with or without a macrolide, for pulmonary Mycobacterium kansasii lasts more than a year. Therefore, shorter treatment duration regimens are required. We used data from 32 Taiwanese patients treated with standard therapy who were followed using repetitive sampling-based sputum *M. kansasii* time-to-positivity in liquid cultures to calculate kill slopes (γ) based on ordinary differential equations and time-to-extinction of each patient’s bacterial burden. The γ was 0.18 (95% confidence interval [CI] = 0.16 to 0.20) log_10_ CFU/ml/day on standard therapy. Next, we identified the *M. kansasii* time to extinction in the hollow-fiber system model of pulmonary M. kansasii disease (HFS-*Mkn*) treated with standard therapy, which was a γ of 0.60 (95% CI = 0.45 to 0.69) log_10_ CFU/ml/day. The γ and time-to-extinction values between the two data sets formed structure-preserving maps based on category theory: thus, we could map them from one to the other using morphisms. This mapping identified a multistep nonlinear transformation factor for time to extinction from HFS-*Mkn* to patients. Next, a head-to-head study in the HFS-*Mkn* identified median time to extinction for standard therapy of 38.7 (95% CI = 29.1 to 53.2) days, isoniazid-rifampin-ethambutol-moxifloxacin therapy of 21.7 (95% CI = 19.1 to 25) days, isoniazid-rifampin-moxifloxacin therapy of 22 (96% CI = 20.1 to 24.5) days, and rifampin-moxifloxacin-tedizolid therapy of 20.7 (95% CI = 18.5 to 29) days. Our transformation-factor based translation predicted the proportion of patients of 90.7 (88.74 to 92.35)% achieving cure with standard therapy at 12 months, and 6-month cure rates of 99.8 (95% CI = 99.27 to 99.95)% for isoniazid-rifampin-ethambutol-moxifloxacin, 92.2 (90.37 to 93.71)% for isoniazid-rifampin-moxifloxacin, and 99.9 (99.44 to 99.99)% for rifampin-moxifloxacin-tedizolid. Thus, rifampin-moxifloxacin-tedizolid and isoniazid-rifampin-ethambutol-moxifloxacin are predicted to be short-course chemotherapy regimens for pulmonary M. kansasii disease.

## Abstract (precis)

Standard therapy (isoniazid, rifampin, and ethambutol), with or without a macrolide, for pulmonary Mycobacterium kansasii lasts more than a year. Therefore, shorter treatment duration regimens are required.

## TEXT

Mycobacterium kansasii pulmonary has been increasing worldwide (1). However, pulmonary *M. kansasii* is still a rare disease (https://rarediseases.info.nih.gov/diseases), which makes it difficult to establish an evidence base for therapeutic decision-making. The Infectious Diseases Society of America (IDSA) recommends a combination of isoniazid (300 mg/day), rifampin (600 mg/day), and ethambutol (15 mg/kg/day), as the standard regimen for pulmonary *M. kansasii*, while the British Thoracic Society (BTS) guidelines recommend daily rifampin, ethambutol, and either isoniazid or a macrolide for at least 12 months after negative sputum conversion (2, 3). In the recently published guidelines, similar to the 2007 guidelines, the treatment recommendation for patients with rifampin-susceptible *M. kansasii* pulmonary disease was a regimen of rifampin and ethambutol in combination with either isoniazid or a macrolide; however, this was a “conditional recommendation, very low certainty in estimates of effect” (2, 4). Due to the lack of randomized controlled trials comparing shorter treatment regimens, the recommendation was to continue the treatment for at least 12 months. Thus, the bottom line, based on both IDSA and BTS guidelines, is that treatment for *M. kansasii* pulmonary disease lasts for more than a year, which is too long, and even then the long-term mortality rate is 24%, which is poor (5–7). Here, we sought to identify potent shorter treatment duration regimens (6 months or less) or short-course chemotherapy regimens.

The hollow-fiber system (HFS) model of tuberculosis (HFS-TB) has been used to design new and shorter-duration pulmonary tuberculosis treatment combination regimens, including those with moxifloxacin and tedizolid (8–10). The HFS has the advantage of allowing repetitive sampling of each system for bacterial burden, much like in patients sputum-based repetitive sampling with quantitative liquid cultures based on the time to positivity (TTP). We have written a set of ordinary differential equations of sputum Mycobacterium tuberculosis burden trajectories in patients on standard therapy, as well as for bacterial burden changes in the HFS-TB, and calculated the time to extinction (TTE) of the entire bacterial burdens, detailed in the methods (11). These two data sets in TB formed a structure-preserving map as defined by category theory (12–14): they could be mapped from one set to the other with a mathematical function that preserved the group operation. With this approach, it is not required that the HFS reproduce the pathology and heterogeneity of the pulmonary disease but only that it can mathematically be mapped by a function that preserves the relationship (15). Given this mathematical property, we could use morphisms to calculate a multistep transformation function to map the time to extinction from the HFS-TB to patients. In TB, this approach correctly predicted cure rates versus duration of therapy in several clinical trials of novel TB regimens (11, 16). Here, we used the same ordinary differential equations and category theory with serial sputum *M. kansasii* TTP in a cohort of Taiwanese patients with pulmonary disease on treatment with IDSA recommended therapy and from HFS of pulmonary *M. kansasii* (HFS-*Mkn*) on standard therapy (17, 18). We then used the findings to identify potential short-course chemotherapy regimens for pulmonary *M. kansasii*.

## RESULTS

### Conversion of time to positivity to CFU.

**FIG 1:**
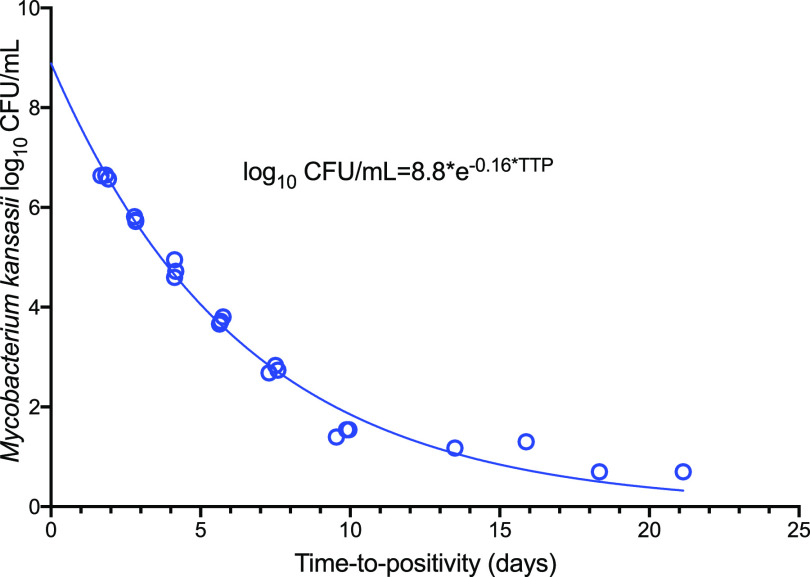
Conversion of TTP to CFU using accrued data from prior work. Shown are the log_10_ CFU/ml versus a TTP of <43 days and above the CFU/ml limits of quantitation. The model was allowed to extrapolate beyond these bounds. The *r*^2^ for the regression was 0.99.

Since the bacterial burden in patient’s sputa was derived from a mycobacterium growth indicator tube (MGIT)-based time to positivity (TTP), our first step was to convert the time-to-positivity values to CFU/ml. First, we utilized data pairs of the *M. kansasii* burden from our various laboratory experiments in which we simultaneously measured the TTP and CFU/ml from the same cultures to identify a formula for converting *M. kansasii* TTP to CFU/ml. Since the MGIT time in protocol was set to 42 days, data from MGIT tubes were flagged as negative growth after 42 days of incubation and were not included in our model, but the model was allowed to extrapolate below these cutoffs. The best conversion formula is shown in Fig. 1, a formula that is virtually the same as what we identified for M. tuberculosis (11). An *r*^2^ value of 0.99 demonstrates a high goodness of fit.

### Clinical description of pulmonary *M. kansasii* patients from Taiwan.

Next, we identified data from 52 patients who had serial sputum samples with MGIT TTP results, based on a study conducted in Taiwan between 2008 and 2014. The following patients were excluded from the modeling: four patients who died within 3 months of initiation of the therapy, three patients who had <2 sputum samples to follow up (no real repetitive sputum culture results), and 13 patients not treated by the standard regimen (treatment regimen included fluoroquinolones or rifabutin replacing rifampin). Thus, the total final number of patients included in the time-to-extinction modeling was 32. The baseline patient demographics, clinical characteristics, drug dose, and time to culture conversion and follow-up are summarized in Table 1. These 32 patients contributed 146 (141 [97%] sputum and 5 [3%] bronchioalveolar lavage [BAL]) culture samples over time. We excluded the BAL samples from further analysis because they are not as practical with regard to repetitive sampling. The median observation period was 687 days, with a maximum follow-up duration of 1,098 days. The median time-to-negative sputum cultures was 130 days (range, 35 to 406 days). Of these 32 patients, two had no culture conversion and thus failed the treatment, and among the remaining 30 patients who showed culture conversion, the 6-month conversion rate was 80% (24/30).

**TABLE 1:** Baseline demographic, clinical, and mycobacteriologic characteristics in 32 patients^*a*^

Parameter	Total (*n* = 32)	Culture conversion (*n* = 30)	No culture conversion (*n* = 2)	*P*
Demographic
Avg age (yr)	58.7 (19–89)	56.2 (19–31)	64.3 (52–75)	0.952
No. (%) male	23 (72)	22 (73)	1 (50)	0.641
Avg wt (kg)	55.6 (31–89)	55.7 (31–89)	53 (39–69	0.997
Clinical
Chest X-ray score	8.2 (3.6)	8.3 (3.7)	7 (1.4)	0.634
Isoniazid (mg/day)	278.1 (69.8)	280 (50.6)	250 (70.7)	0.430
Rifampin (mg/day)	543.8 (135.8)	550 (99.1)	450	0.170
Ethambutol (mg/day)	935.2 (267.3)	950.8 (700)	700 (141.4)	0.112
Treatment duration (days)	392.3 (137.5)	395.9 (140.5)	339 (82.27)	0.579
Follow-up duration (days)^*b*^	686.7 (326.8)	674.9 (334.3)	862.5 (62.9)	0.441
Sputum no. during follow-up	3.5 (1.5)	3.3 (1.52)	5.0 (1.4)	0.142
Specimen type, no. (%)
Sputum	28 (87.5)	26 (86.7)	2 (100)	1.000
BAL	4 (12.5)	4	0
Time (days)
Baseline time to positivity	36.0 (12.9)	35.1 (12.1)	48.5 (23.3)	0.158
Time to culture conversion	130.3 (89.7)	130.3 (89.7)

a Ranges or standard deviations are indicated in parentheses where applicable.

b The follow-up duration covers from the end date of treatment to the last follow-up date.

**FIG 2:**
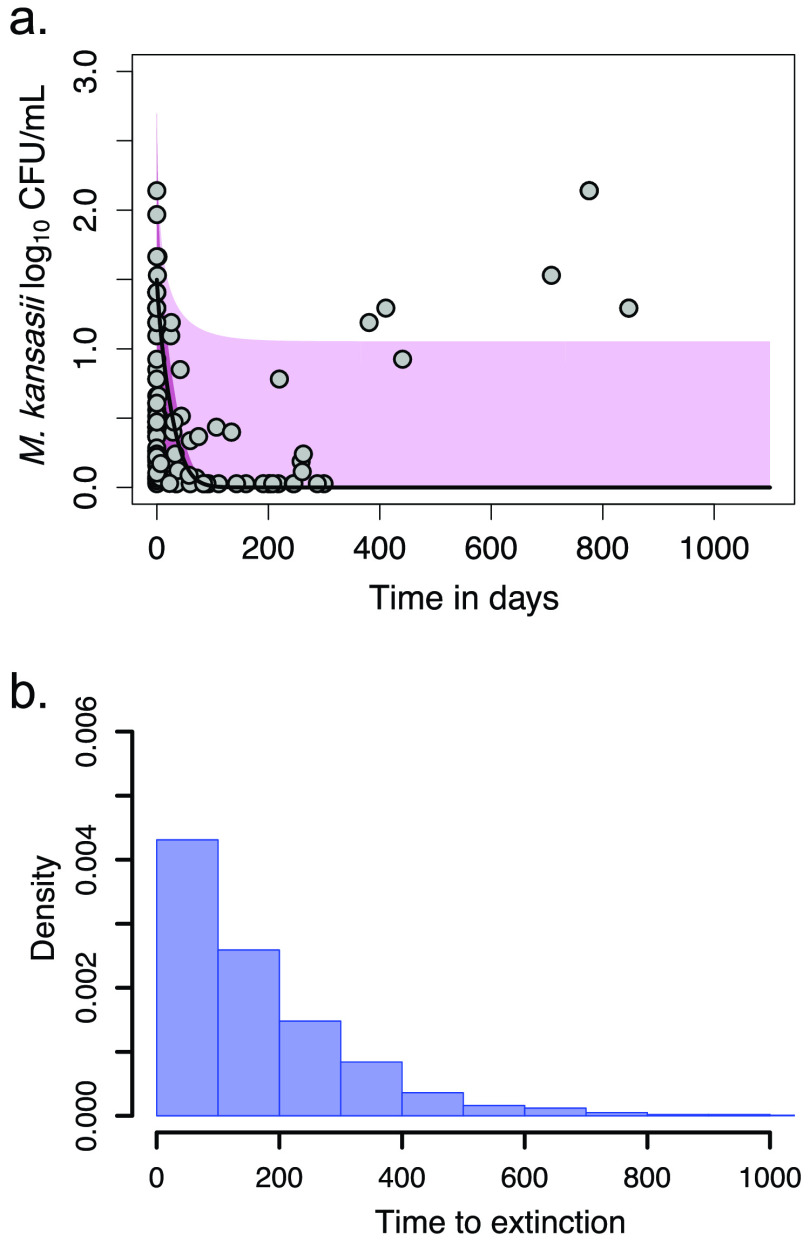
Bacterial burden change and time to extinction in 32 patients. The curve represents median values, whereas the shaded area depicts the upper and lower bounds of the 95% CI values. (a) Trajectories of M. kansasii log_10_ CFU/ml in serial sputum samples (141 data points) collected in 32 patients treated with the standard therapy. Two patients did not achieve culture conversion. (b) Distribution of *M. kansasii* population times to extinction in patients based on the trajectories in panel A. In patients who did not sputum convert, this was predicted to be beyond 1,000 days.

Next, we converted the TTP from patient’s sputa to CFU/ml, using the formula in Fig. 1. We then used the ordinary differential equations discussed in detail in Materials and Methods to identify *M. kansasii* CFU/ml-based kill rates in patient’s sputa. Figure 2a shows the *M. kansasii* log_10_ CFU/ml decline in the patient sputa, including those who did not sputum convert, and demonstrates a slow decline in bacterial burden of up to 3 years. We identified a γ (kill rate) of 0.18 (95% confidence interval [CI] = 0.16 to 0.20) log_10_ CFU/ml/day on standard therapy, and a *K_max_* of 0.70 (95% CI = 0.26 to 0.98), where *K_max_* is the carrying capacity (i.e., the maximal bacterial population possible in lungs). Based on these slopes, the *M. kansasii* population time to extinction, defined as the time to the sputum bacterial burden falling below 10^−2^ CFU/ml (see Materials and Methods for the derivation of this definition), and its distribution in the 32 patients were calculated as shown in Fig. 2b. The figure shows a median time to extinction of 151.5 (95% CI = 48.1 to 1,069.1) days, when the two patients who did not sputum convert are not considered; otherwise, the average time to extinction would stretch to 2.7 years. This means that total bacterial eradication (i.e., the extinction of the bacterial population) was achieved after a median of 22 weeks of therapy; as shown in Fig. 2b, these values were not normally distributed and were widely variable, stretching from 6.9 to 56 weeks. This is consistent with clinical observations of treatment duration on standard therapy.

### Mapping time to extinction on standard therapy from the HFS-*Mkn* to patients.

**FIG 3:**
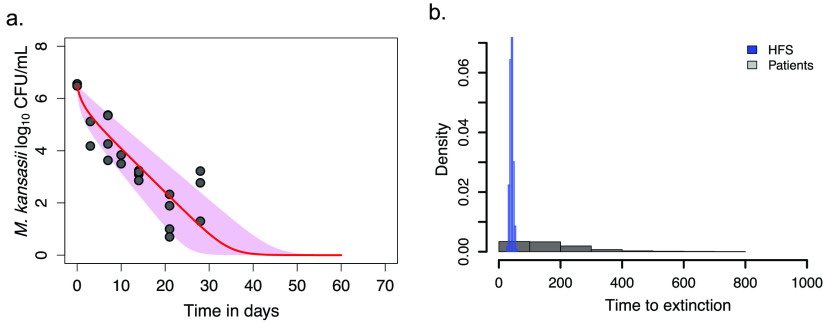
Bacterial burden change and times to extinction on standard therapy in HFS-*Mkn*. The curve represents median values, whereas the shaded area depicts the upper and lower bounds of the 95% CI values. (a) Kill slopes of the *M. kansasii* burden on standard therapy in HFS-*Mkn*. Given the steep γ slopes, the time scale of the *x* axis used for the HFS-*Mkn* is different from that in patients in Fig. 1 and stretches only to 70 days. (b) Comparison of distributions of two sets of times to extinction. The HFS-*Mkn*-derived data in blue and the clinic-derived data from Fig. 1 in gray show a narrower distribution and different shape of distribution for HFS-*Mkn*. Thus, the transformation or mapping between the two data sets was nonlinear, nonsymmetrical, and multistep.

Next, we performed HFS-*Mkn* work with replicates of nontreated systems and replicates of systems treated by the same standard therapy as the patients in Taiwan in several published studies (18, 19). Bacterial burden readouts from HFS-*Mkn* units treated with standard therapy versus nontreated were modeled using the exact ordinary differential equations used in patient sputa and identified the log_10_ CFU/ml kill slopes shown in Fig. 3a. The HFS-*Mkn*-derived γ slope on standard therapy was 0.60 (95% CI = 0.45 to 0.69) log_10_ CFU/ml/day, the bacterial carrying capacity was 8.25 (95% CI = 7.75 to 8.48) log_10_ CFU/ml. The HFS-*Mkn*-derived times to extinction and distributions are shown in Fig. 3b, which demonstrates an HFS-*Mkn* time to extinction of 40.7 days (95% CI = 29.7 to 51.9 days). Figure 3b also compares patient data and shows that the HFS-*Mkn* time-to-extinction distribution and range differ from that derived in patient sputa and is narrower because HFS*-Mkn* studies have a relatively uniform bacterial burden at the start of therapy, whereas patients have large variation in bacterial burden at the start of treatment and lesion heterogeneity.

Comparison of the patient sputa data set and the HFS-*Mkn* data set revealed that while the shapes and magnitudes of the time-to-extinction probability distributions differed, they nevertheless formed a structure-preserving map, that is, a morphism, as defined by category theory. The data sets could be mapped by a nonlinear transformation factor that was a vector of 3.73 (95% CI = 1.62 to 20.6) for the distribution of HFS-*Mkn* times to extinction to patients (Table 2). The upper-bound translation factor of 20.6 indicates that the HFS-*Mkn* predicted treatment endpoint of 51.9 days should be stretched with a factor of that magnitude to predict the corresponding clinical outcome; however, a time-to-extinction value of 29.7 days in the HFS-*Mkn* models would be transformed with a factor of magnitude of only 1.62 to estimate the lower bound of treatment duration in patients. Thus, the translation was not scaling as a single scalar value but was multistaged, arising from the fact that patients have lesion heterogeneity and large initial bacterial burden variability as opposed to the narrow burden and slope variability in the HFS-*Mkn*.

**TABLE 2:** Transformation factors and transformed time to extinction in the HFS-*Mkn* and patients^*a*^

Parameter	Lower bound of 95% CI	Median	Upper bound of 95% CI
Transformation factor	1.62	3.73	20.6
Time to extinction in HFS-*Mkn* on standard therapy	29.7	40.7	51.9
Time to extinction in patients on standard therapy	48.11	151.40	1,069.14

a Transformation factors for mapping HFS-*Mkn* data to humans. The median is used as the best estimate of the time to extinction in the HFS-*Mkn* and to transform to patients treated using the same regimen but the confidence intervals stretch asymmetrically.

### Examination of novel regimens versus standard therapy in the HFS-*Mkn*.

**FIG 4:**
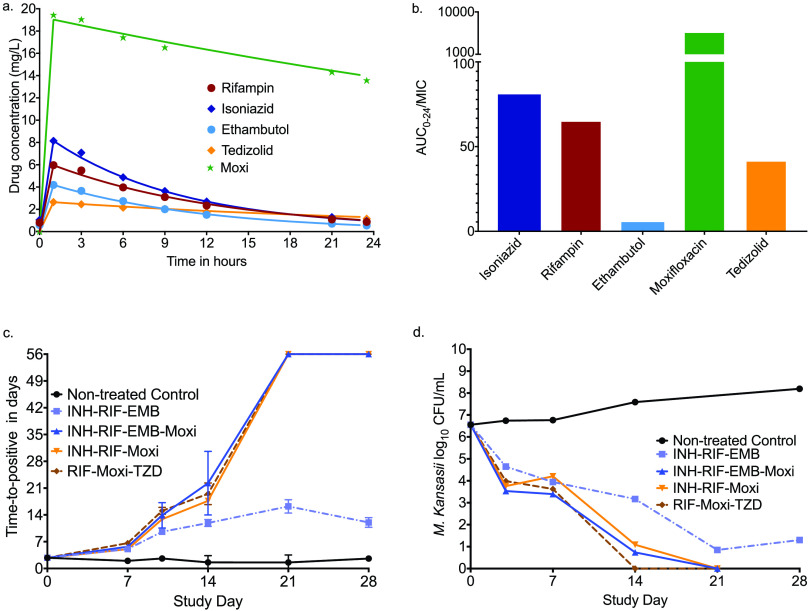
Concentration- and bacterial burden-versus-time curves in the HFS-*Mkn*. Data points are mean values from hollow fiber replicates; error bars indicate the standard deviations. (a) Drug concentrations at each time point in all hollow-fiber systems which received the specified drug. The regression lines are pharmacokinetic model-predicted concentration-time profiles. (b) The AUCs achieved in the hollow fiber are meant to approximate those at site of infection, in this case lung cavitary lesions (15, 21–24). The moxifloxacin AUC_0–24_ of 355 ± 7 mg⋅h/liter is high, but actually similar to the median achieved in tuberculosis caseum, for example (21). Isoniazid, rifampin, and tedizolid had MICs of 1 mg/liter, moxifloxacin had an MIC of 0.06 mg/liter, and ethambutol had an MIC of 8 mg/liter, resulting in the AUC/MIC ratios shown. (c) Based on the time-to-positivity (TTP) readout, which increases with decreasing bacterial burden, the standard therapy combined with moxifloxacin regimen achieved negative cultures by day 21, followed by rifampin, moxifloxacin, and tedizolid was determined. (d) On the other hand, based on the CFU/ml readout, the rifampin-moxifloxacin-tedizolid regimen achieved negative status first. The nontreated controls (negative controls) grew based on both time-to-positivity and CFU/ml readouts.

Given this ability now to translate kill slopes from the HFS-Mkn to patients, we performed a head-to-head comparison of several regimens versus the ATS/IDSA-recommended none-macrolide regimen in the HFS-*Mkn* (2, 4). A comparison of this regimen to the macrolide-containing BTS regimen in the HFS-Mkn has been published (20). The concentration-time profiles of each drug achieved in the HFS-*Mkn* were as shown in Fig. 4a. The HFS-*Mkn* AUC_0–24_/MIC exposures shown in Fig. 4b can be achieved at the site of infection in lung lesions and/or epithelial lining fluid at standard doses and clinically tolerated higher doses for each drug (15, 21–24). Based on the broth microdilution method, the *M. kansasii* strain used in the HFS-*Mkn* had isoniazid, rifampin, and tedizolid MICs of 1.0 mg/liter, while that for ethambutol was 8 mg/liter and that for moxifloxacin was 0.06 mg/liter. The time-kill slopes achieved by regimens that contained these exposures are shown in Fig. 4c for the time to positivity and in Fig. 4d for the CFU/ml readout. In both readouts, all experimental regimens had negative cultures starting on day 21, but not in the standard therapy arm.

**FIG 5:**
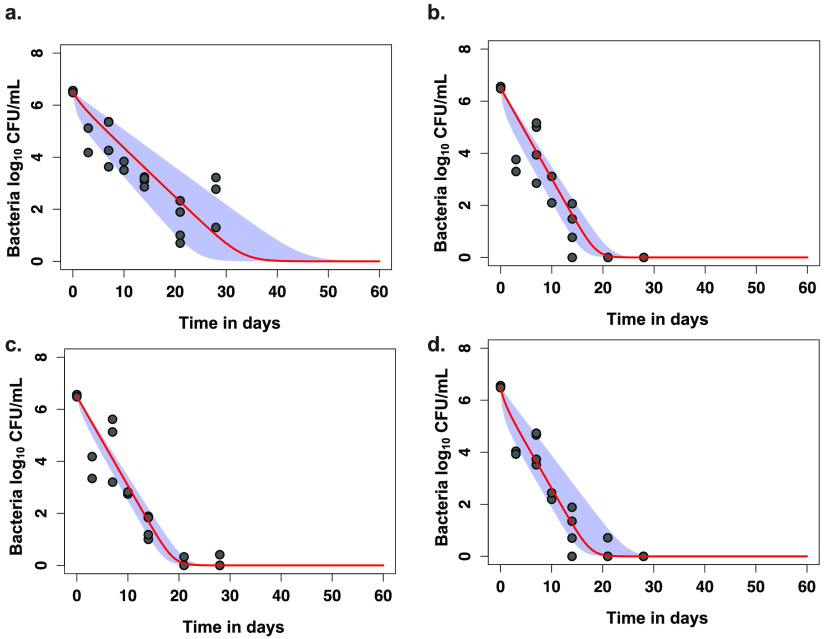
Ordinary differential equation and time to extinction in the hollow-fiber system. Our ordinary differential equation integrates both time-to-positivity and CFU/ml readouts from the HFS-*Mkn* into one model. The circles are observed bacterial burden at different sampling time points, the line is the median γ slope, and the shaded areas are the upper and lower bounds of the 95% CI values for different regimens, from which are calculated the median time to extinction (TTE). (a) The TTE for standard therapy was 38.7 (95% CI = 29.1 to 53.2) days. (b) The same for standard therapy plus moxifloxacin was 21.7 (95% CI = 19.1 to 25) days. (c) The TTE for isoniazid-rifampin-moxifloxacin was 22 (96% CI = 20.1 to 24.5) days. (d) The TTE in the HFS-*Mkn* for the rifampin-moxifloxacin-tedizolid was 20.7 (95% CI = 18.5 to 29) days.

Next, we applied our ordinary differential equations and identified the trajectories, γ slope results, and times to extinction in the HFS-*Mkn* (Fig. 5). Figure 5 shows that all experimental regimens had statistically lower time-to-extinction compared to standard therapy, which was due to the higher γ slopes.

**FIG 6:**
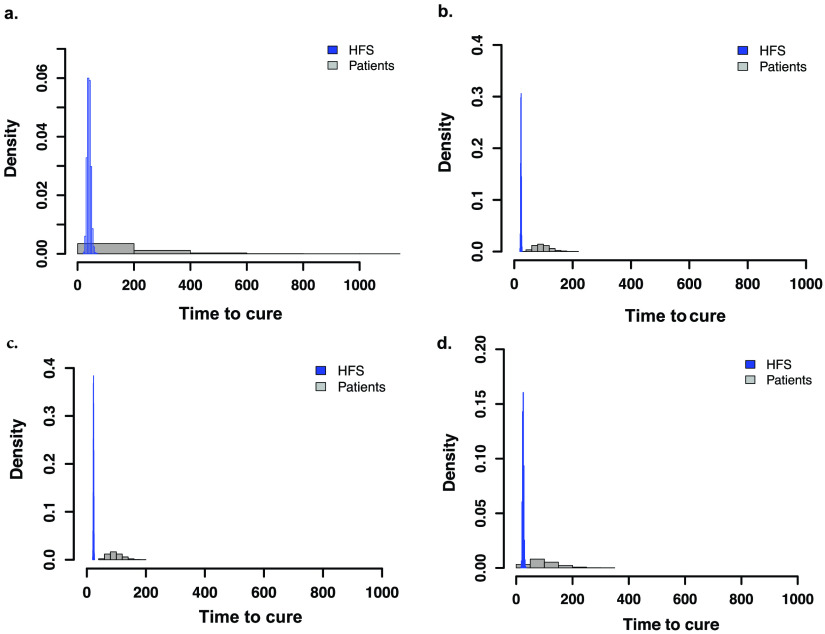
Translation of time to extinction from hollow fiber to the clinic using a multistep nonlinear transformation factor. The transformation time to extinction from HFS-*Mkn* (blue) to that predicted in patients (gray) for each regimen using Latin hypercube sampling of 1,000 patients each. Median and 95% CI values are shown. (a) Translation for standard therapy predicts a time to extinction or minimum duration of therapy of 144.35 (95% CI = 47.14 to 1,095.92) days in patients on standard therapy. (b) Addition of moxifloxacin reduces the time to 80.98 (95% CI = 30.94 to 515) days. (c) In the same regimen, but without ethambutol, the median was 82.06 (95% CI = 32.56 to 505.7) days in patients, so that contribution of ethambutol is meager. (d) Replacement of isoniazid with tedizolid for the RIF-Moxi-TZD regimen improves both the median and upper bounds: 77.21 (95% CI = 29.97 to 597.4) days in the patients, which is the shortest of all four regimens.

The time-to-extinction distributions from the HFS-*Mkn* for each regimen were translated using the transformation factor (*T_f_*) derived earlier (Table 2) from the HFS-Mkn to 1,000 patients per regimen (Fig. 6). Figure 6a shows the time-to-extinction predictions for standard therapy of 144.35 (95% CI = 47.14 to 1,095.92) days in patients, which harmonizes well with the time to extinction of 151.5 (95% CI = 48.1 to 1069.1) days that we identified in 32 Taiwan patients (see Fig. S1 in the supplemental material); thus, our translation step was successful. Figure 6b to d show the predictions for the experimental regimens.

**FIG 7:**
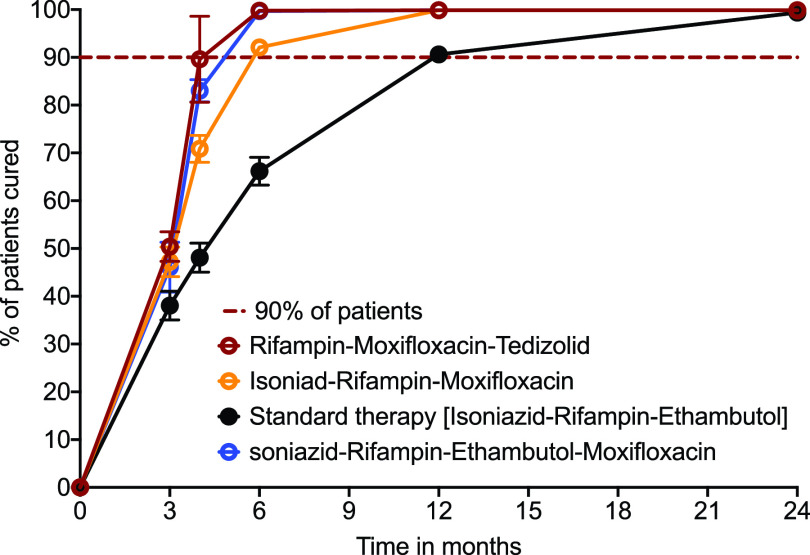
Predicted time to extinction in 1,000 patients treated with different regimens. Error bars indicate the 95% CI values, and symbols indicate the median percent patients that have *M. kansasii* populations/burden that have reached extinction. Standard therapy 95% CI lower-bound values fall below 90% at 12 months, and thus therapy needs to be prolonged beyond 12 months, consistent with current recommendations. Two regimens, rifampin-moxifloxacin-tedizolid and standard therapy plus moxifloxacin, achieved *M. kansasii* extinction prior to 6 months, with the isoniazid-rifampin-moxifloxacin lower credible bounds achieving this in >90% of patients at 6 months.

All relapse and therapy failure (as opposed to reinfection) occurs because of failure to achieve bacterial population extinction, by definition. However, not all patients who fail to reach extinction will fail or relapse. The proportions of patients whose bacterial populations reached extinction at different time points, which is defined as the minimum duration of therapy associated with relapse-free cure in the clinical predictions, are shown in Fig. 7. The standard regimen achieved a time to extinction in >90% of patients in the lower credible interval bounds at 24 months. Starting with the shortest duration of therapy predicted in patients, the ranking of the regimens was as follows: rifampin-moxifloxacin-tedizolid therapy is equal to standard therapy plus high-dose moxifloxacin, with isoniazid-rifampin-moxifloxacin ranking third, followed by standard therapy as the worst. This ranking could be used to determine which regimen to test first in patients.

## DISCUSSION

In routine patient care for pulmonary *M. kansasii*, as in patients with TB, sputum is routinely collected. Liquid culture readouts of time to positivity in the semiautomated MGIT system allow us to quantify the bacterial burden and conversion to CFU/ml. This bacterial burden data versus time allowed us to apply our set of ordinary differential equations to patient sputum. Thus, the first major contribution of our current work is that we mapped the *M. kansasii* kill trajectories and time to extinction in patients on standard therapy, using routinely collected clinical data (17). The kill slopes (γ slopes) derived from patients on an ATS/IDSA-recommended therapy regimen demonstrated slow kill rates, which explains why a longer therapy duration is needed with the standard regimen. Specifically, the kill rates and time-to-extinction distribution mean that the decision to treat for >12 months as recommended in the guidelines has a sound pharmacodynamic basis (2–4). This also means that our ordinary differential equations and time-to-extinction models yield parameters consistent with clinical therapeutic events and are therefore useful in designing different durations of therapy for patients with pulmonary *M. kansasii*. These slopes can now be used as clinical pharmacodynamic outcomes, since they are determinative of time to cure and duration of therapy.

Given the nature of repetitive sampling in the HFS-*Mkn*, which parallels that in patients, the same equations and models allowed derivation of slopes and time-to-extinction data sets in both patients and HFS-*Mkn*. Mathematically, these formed structure-preserving maps and could thus be mapped back and forth using morphism functions. This allows a straightforward derivation of a quantitative translation factor from the HFS-*Mkn* to patients. In other words, we now have a way to relate what a kill slope in the HFS-*Mkn* means to kill slopes in patients, which is crucial to designing shorter therapy durations. The mapping factor we derived here allows us to translate trajectories from the HFS-*Mkn* to patients to identify minimum duration of therapy associated with high cure rates for any *M. kansasii* treatment regimen to be tested in the future.

With regard to the three new experimental regimens, we found that the strategy of adding moxifloxacin at the previously identified optimal dose for *M. kansasii* to the current standard of therapy resulted in predicted improved efficacy and a possibility of considerably shorter therapy duration, that is, only (6/24) 25% that of the standard therapy (19). The recent treatment recommendations (4) acknowledge that while rifamycin-based drug regimens are effective, the best companion drugs are yet to be determined and that addition of fluoroquinolone could likely lead to successful treatment outcome. Elsewhere, we also compared the macrolide-containing regimen for *M. kansasii* to moxifloxacin-containing regimens, and its HFS-*Mkn* kill slopes, while better than macrolide-free regimens, were poorer than one of the moxifloxacin-based regimens proposed here (20). In our patients from Taiwan, there were two patients who also received moxifloxacin 400 mg per day in addition to standard therapy (and were thus not part of the 32 patients for model derivation). The median time-to-culture conversion (and not time to extinction) in these two patients on standard dose moxifloxacin plus standard therapy was 57 days, which is 44% shorter than the 130 days in our 32 patients on standard therapy, whose data we modeled. Our regimen in the head-to-head HFS-*Mkn* had a moxifloxacin dose equivalent to 800 mg/day. Similarly, the tedizolid-moxifloxacin-rifampin combination regimen shows promise. Increasing the rifampin dose for each of these regimens from 10 mg/kg to the 45 mg/kg/day currently being examined in tuberculosis clinical trials (shown to be safe) could further shorten therapy duration of the proposed regimens to possibly 4 months (15, 25). However, the optimal tedizolid dose for the combination therapy for *M. kansasii* is unknown and could also further shorten therapy duration. In addition, our current model did not examine adverse events, which are common with oxazolidinones.

Finally, we propose use of kill trajectories (γ slopes) as clinical pharmacodynamic outcomes measure, since they are determinative of the time to cure and the duration of therapy. This is because *M. kansasii* is an orphan disease, and it is acknowledged that large randomized controlled trials that examine relapse will be logistically difficult. We propose a new clinical-trial approach that utilizes the TTE parameter in patients in pragmatic trials to test the promising regimens we found for both macrolide-containing and macrolide-sparing regimens or any other new regimens. We propose treating cohorts of patients with these HFS-*Mkn*-derived novel regimens for about 6 months while collecting weekly sputum for bacterial burden to determine whether the sputum trajectory indicates that they have reached extinction. Patients who still have positive culture at 6 months (using the 5-month time point result) will be deemed to have failed and will be started on standard therapy (or continued on standard therapy) for the currently recommended durations. Patients who have reached *M. kansasii* population extinction can then have therapy stopped and be closely monitored for relapse with regular radiography and sputum cultures for 2 years after therapy.

Our study is not without limitations. First, there were relatively limited clinical data from patients to calculate the kill slopes for different growth rates of *M. kansasii*. Second, the clinical data were retrospectively collected, without standard follow-up protocols. Thus, the slopes and TTE values in patients will need to be further refined with prospectively collected clinical data. Third, all of the patients included in the model development were not HIV infected. Thus, our findings may not be generalized to the patients with dual HIV-*M. kansasii* infections. Finally, since a TTP of >43 days was not included in the model for TTP-CFU conversion, this could result in a biased data set. However, given even the good fit for the noncensored data, the bias is expected to be minimal.

In summary, we developed a mathematical translational framework to predict the therapy duration in patients based on the preclinical findings. Given that *M. kansasii* pulmonary disease is a rare disease and large randomized controlled trials are rare, our translational framework may supplement RCTs. Our study is a proof of concept that an *M. kansasii* pulmonary infection could be treated in less than a year or possibly in 6 months with a combination of rifampin, moxifloxacin, and tedizolid.

## MATERIALS AND METHODS

### Conversion of *M. kansasii* TTP to CFU/ml.

We followed steps used for conversion of M. tuberculosisTTP to CFU/ml described in detail in the original publication (11). The function *F*(*x*) for the bacterial burden in log_10_ CFU/ml was expressed using the exponential function (*e*):

(1) $$F(x)=\alpha e^{-\beta x\;+\;\gamma }$$

whereby the function γ was either set at zero (model 1) or with various nonzero values based on therapy duration (model 2), where *x* is the bacterial burden expressed as TTP in days, α is the log_10_ CFU/ml that corresponded to the TTP theoretical value associated with no growth, β is the term in the exponent explaining the conversion of increasing TTP values to CFU/ml, and γ is adjusted for the level of variation in the conversion. The initial values used were similar to those identified in the models for M. tuberculosis (11), another slowly growing mycobacterium, but the final model was allowed to calculate the final values. Values below TTP of 43 days and below the CFU/ml limits of quantitation were censored or not used, but the model was allowed to extrapolate beyond these bounds.

### Ordinary differential equation application.

In patients’ sputa and in the hollow fiber system, we utilized a system of ordinary differential equations 2 and 3, originally derived for M. tuberculosis subpopulations (11):

(2) $$\frac{dB_{f}}{\mathrm{dt}}=\left(1\;-\;\epsilon_{f}\right)r_{f}B_{f}\left(1\;-\;\frac{B_{s}\;+\;B_{f}}{K_{\max}}\right)\;-\;\gamma_{f}B_{f}$$

(3) $$\frac{dB_{s}}{\mathrm{dt}}=\left(1\;-\;\epsilon_{s}\right)r_{s}B_{s}\left(1\;-\;\frac{B_{s}\;+\;B_{f}}{K_{\max}}\right)\;-\;\gamma_{s}B_{s}.$$

where *B_f_* is the CFU/ml of fast-replicating bacteria, *B_s_* is the CFU/ml of semidormant/nonreplicating (slow) bacteria, *t* is the time, *r_f_* and *r_s_* model the rate of replication of the fast- and slow-replicating bacteria, *K_max_* is the maximum bacterium-carrying capacity, and *γ_s_* and *γ_f_* are the antibiotic regimen kill slopes for slow- and fast-replicating bacteria, respectively. We tested for *M. kansasii* as two separate populations based on growth rates (equations 1 and 2) or as a population with a single growth rate (one equation with one bacterial growth rate), and then we chose the best model for *M. kansasii*. The systems of equations 1 and 2 are linearized through evaluating the Jacobian value (J) as detailed in the original publication (11). This led to the solution that sterilization of the bacterial population in the lung is achieved when sputum bacterial burden falls below 10^−2^ (*B_f_* + *B_s_* < 10^−2^) (11). The definitions of a linear map/morphism, inverse transformation/mapping, and a composite linear map, as well as the computation of the mapping transformation factor (*T_f_*) of the bacterial trajectories, γslopes, and times to extinction between the HFS-*Mkn* and patients were identical to those for M. tuberculosis, described in detail in the original paper (11).

### Clinical data collection.

A retrospective clinical study, approved by the respective institutional ethic committees (NTUH-REC-201508017RIND and KMUHIRB-SV[I]-2015200266), was conducted in six hospitals in Taiwan between 2008 and 2014 (17). Patient demographics and HIV infection status were retrieved from the case report forms (CRF) in the study records. The CRFs are shown in the methods provided in the supplemental material. Patients with pulmonary *M. kansasii* isolates who fulfilled the microbiological criteria for disease and had serial sputum samples collected were identified. Symptoms, signs, medical history, and other laboratory data were also reviewed from the hospital charts to exclude diagnoses of diseases other than *M. kansasii* pulmonary disease.

### Determination of MIC and concentration-response studies in test tubes.

The MICs of the drugs were determined using the broth microdilution method (26). Briefly, *M. kansasii* cultures were grown for 4 days to obtain the logarithmic-phase culture, and the inoculum was prepared to get a bacterial density of ∼1.5 × 10^5^ CFU/ml. The concentration ranges for each of the drugs are presented in Table 3. The experiment was set in 96-well plates in triplicate for each concentration, including the untreated control. The cultures were incubated for 7 days, and plates were inspected for the presence of a bacterial pellet. The drug concentration that completely inhibited the *M. kansasii* growth (i.e., the absence of a bacterial pellet) was recorded as the MIC. Experiments were performed twice.

**TABLE 3:** Concentration range of each drug tested for MICs

Drug	Concn range (mg/liter) tested
Isoniazid	0, 0.12, 0.25, 0.5, 1, 2, 4, 8, 16, 32
Rifampin	0, 0.03, 0.06, 0.12, 0.25, 0.5, 1, 2, 4
Ethambutol	0, 1, 2, 4, 8, 16, 32, 64
Moxifloxacin	0, 0.6, 0.12, 0.25, 0.5, 1, 2, 4, 8, 16, 32
Tedizolid	0, 0.6, 0.12, 0.25, 0.5, 1, 2, 4, 8

### HFS-*Mkn* studies.

The laboratory strain *M. kansasii* ATCC 12478 and the human monocyte-derived THP-1 cell line (ATCC TIB-202) were used in all HFS-*Mkn* experiments, with culture medium and conditions as reported previously (18, 19). The HFS-*Mkn* has been described in detail previously (18, 19). Briefly, 20-ml portions of *M. kansasii*-infected THP-1 cells were inoculated into the peripheral compartment of 10 HFS-*M. kansasii* units, which were then treated with different daily combination regimens using human lung concentration-time profiles for each antibiotic: (i) a human-equivalent standard regimen of isoniazid at 300 mg/day, rifampin at 600 mg/day, and ethambutol at 900 mg/day, (ii) a standard regimen plus moxifloxacin at 800 mg/day as identified (19) in HFS-*Mkn* in the past, (iii) isoniazid at 300 mg/day plus rifampin at 600 mg/day plus moxifloxacin at 800 mg/day, (iv) rifampin at 600 mg/day plus moxifloxacin at 800 mg/day plus tedizolid at 200 mg/day, and (v) untreated controls (19). There were two replicate HFS-*Mkn* units per regimen. We mimicked a serum 3-h half-life for isoniazid and rifampin and 12-h half-life for ethambutol, moxifloxacin, and tedizolid (10, 19, 27, 28). Samples for drug concentrations, THP-1 cells, and *M. kansasii* burden (CFU/ml and time to positivity) were collected as detailed in the supplemental material.

### Software and statistical analysis.

For TTP to CFU/ml conversion, all model fitting was done in R, but data were transferred to GraphPad Prism for graphing purposes (29). ADAPT II (30) was used to perform the pharmacokinetic analysis using the model parameters and methodologies described in detail in our previous publications for each drug (8, 28, 31, 32). The measured concentrations were used to calculate the *C*_max_, the AUC_0–24_, and the *C*_max_/MIC and AUC_0–24_/MIC ratios. An inhibitory sigmoid *E*_max_ model was used to determine the relationship between the drug concentration and the bacterial burden (GraphPad v7.01). Linear regression was used to calculate the kill rate and with the experimental regimens.

## Supplementary Material

Supplemental file 1
