# Supplementary material for: Novel Short-Course Therapy and Morphism Mapping for Clinical Pulmonary Mycobacterium kansasii
Source: Antimicrob Agents Chemother. 2021 Apr 19;65(5):e01553-20. doi: 10.1128/AAC.01553-20 (PMC8092872; doi:10.1128/AAC.01553-20)
Supplement: Supplemental file 1 [file AAC.01553-20-s0001.pdf]

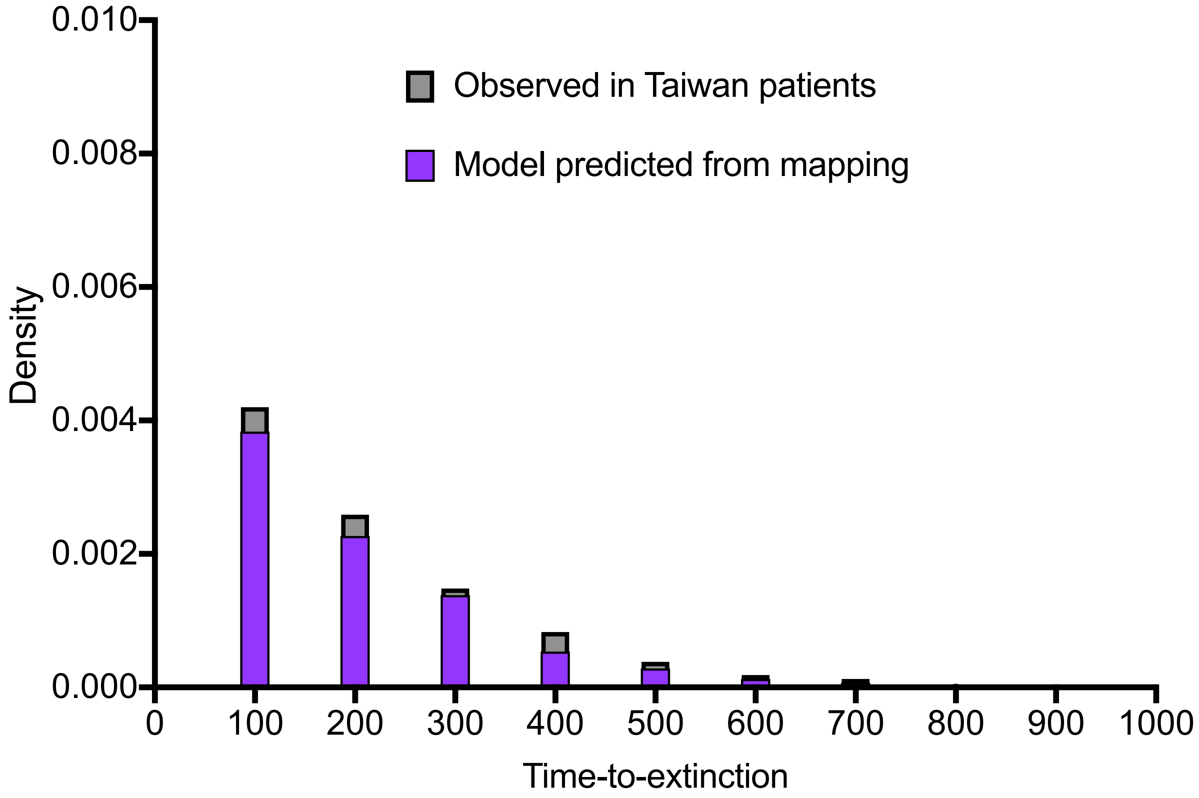

Figure S1. Overlay of time-to-extinction identified in patients and that modeled from hollow fiber system model data. The data in gray is the same as was observed in patients from Taiwan in Figure 2b, while that in grape color was modeled from the standard therapy regimen in the hollow fiber system model studies for head-to-head comparisons with experimental regimens that was then translated to patients [predicted]. The distributions of time-to-extinction are similar, which means that our modeling worked well.
